# Applicability of a Web-based 24-hour Dietary Recall Tool for Japanese Populations in Large-scale Epidemiological Studies

**DOI:** 10.2188/jea.JE20220071

**Published:** 2023-08-05

**Authors:** Yoshie Hose, Junko Ishihara, Ayaka Kotemori, Misako Nakadate, Sachiko Maruya, Junta Tanaka, Hiroshi Yatsuya, Atsuko Aoyama, Chifa Chiang, Tsuneo Konta, Takamasa Kayama, Yoshiyuki Ueno, Manami Inoue, Norie Sawada, Shoichiro Tsugane, Ribeka Takachi

**Affiliations:** 1 Graduate School of Environmental Health, Azabu University, Kanagawa, Japan; 2 Department of Food and Life Science, Azabu University, Kanagawa, Japan; 3 Department of Food Science and Nutrition, Faculty of Human Life and Environment, Nara Women’s University, Nara, Japan; 4 Department of Health Promotion Medicine, Niigata University Graduate School of Medical and Dental Sciences, Niigata, Japan; 5 Department of Public Health, Fujita Health University School of Medicine, Aichi, Japan; 6 Department of Public Health and Health Systems, Nagoya University Graduate School of Medicine, Nagoya, Japan; 7 Nagoya University of Arts and Sciences, Nissin, Aichi, Japan; 8 Department of Public Health and Hygiene, Yamagata University School of Medicine, Yamagata, Japan; 9 Epidemiology and Prevention Group, Center for Public Health Sciences, National Cancer Center, Tokyo, Japan; 10 Department of Food Science and Nutrition, Nara Women’s University Graduate School of Humanities and Sciences, Nara, Japan

**Keywords:** web-based 24-hour dietary recall, dietary assessment, cohort study, calibration, applicability

## Abstract

**Background:**

Recent innovations in information and communication technology have made it possible to assess diet using web-based methods; however, their applicability in the general population remains unclear. Hence, we aimed to examine the applicability of a web-based 24-hour dietary recall (24HR) tool to large-scale epidemiological studies by determining the sampling rate and characteristics of randomly selected participants from a Japanese cohort study.

**Methods:**

In total, 5,013 individuals were recruited from a cohort of 21,537 individuals, and 975 agreed to participate in this study. The participants selected either self-administered web-based dietary 24HR (self-administered 24HR) or interviewer-administered telephone-based 24HR (interviewer-administered 24HR) as the method for the dietary assessment and answered questions regarding the acceptability of the system.

**Results:**

The response rate of the 975 participants was 19.4%, corresponding to approximately 4.5% of the total study sample. About half of them chose the self-administered 24HR (46.9%). The median time required for the self-administered and interviewer-administered 24HR was 25 and 27 minutes, respectively. In the self-administered 24HR, older people, regardless of sex, tended to require a longer time, and approximately 60% of the participants rated the ease of use of the system as “somewhat difficult” or “difficult.”

**Conclusion:**

Characteristics of the participants in this study were not systemically different from those of the entire study sample. Improvements in the approach to entering cooking details and the dish name selection may be necessary for better acceptability in order to be accepted as a self-administered dietary recall tool.

## INTRODUCTION

Information obtained through diet and nutrition assessment is the basis for establishing evidence-based policy for preventing disease and improving public health. In large-scale epidemiological studies, food frequency questionnaires (FFQs) are commonly used.^1^ However, compared with other tools used to assess dietary intake, FFQs tend to have large measurement errors.^2^

Recent innovations in information and communication technology have made it possible in various countries to assess diet using web-based 24-hour dietary recall (24HR) tools.^3^^–^^13^ EPIC-Soft collects data using the 24HR method and can be used to calibrate measurement errors of FFQs.^14^ Web applications and software programs enable efficient data collection and manipulation.

Japanese diets are complex and involve mixed dishes, and the variety of combinations makes it difficult to investigate meals on a food-by-food basis. This may also cause relatively low validity of the FFQ among the Japanese population.^15^ To overcome the complexity and measurement errors of investigation of the Japanese diet, a web-based automated food assessment tool that uses the 24HR method, the Automated Web-based assessment System Using Recipe-Data for Japanese (AWARDJP), was developed.

This study aimed to investigate the estimated dietary intake using the AWARDJP for the calibration of the measurement error of the estimated intake using the FFQ in this population. Towards this goal, we examined the applicability of the AWARDJP in large-scale epidemiological studies by determining the sampling rate and characteristics of randomly selected participants (from the entire study sample) in the Japan Public Health Center Next Generation Prospective Study^16^ (JPHC-NEXT) and other cohorts that use the same FFQ as JPHC-NEXT. Furthermore, we clarified the differences in the acceptability of the AWARDJP by the response method used in the dietary assessment.

## METHODS

### Study participants

**Figure 1.**
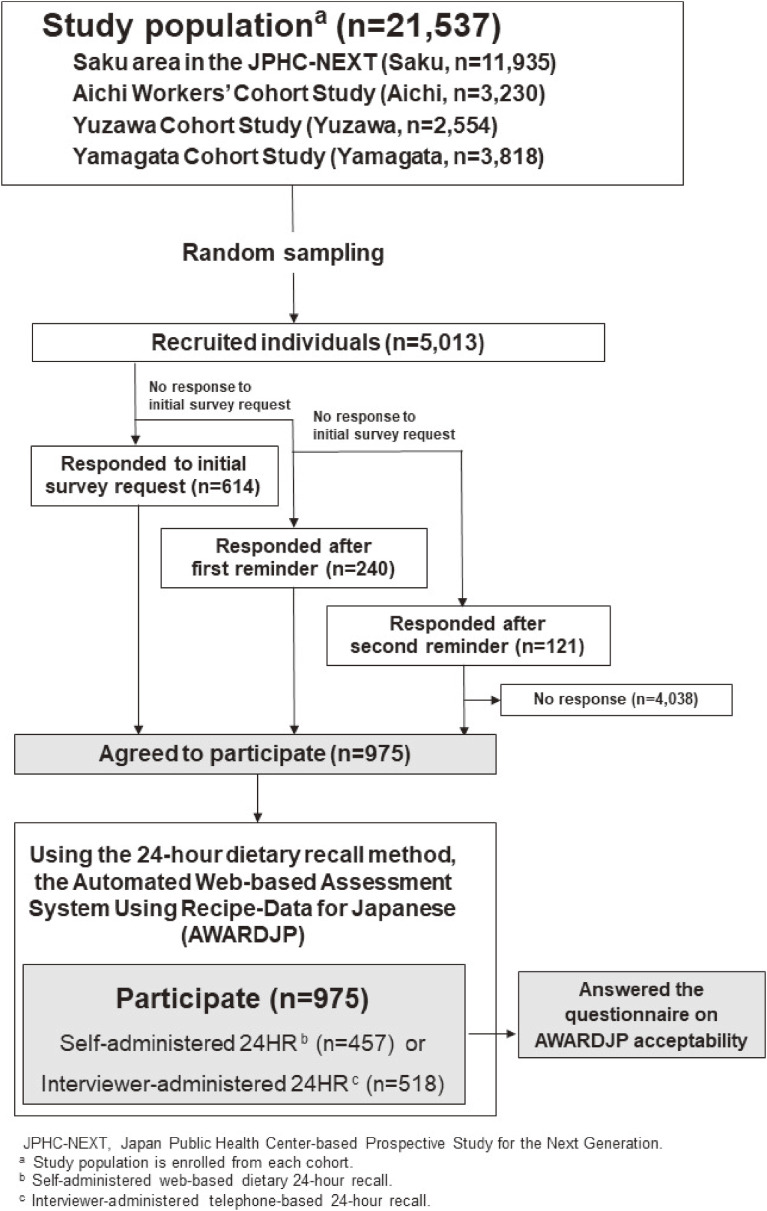
Flow of data collection

Study participants were recruited from the following four cohort studies: the Saku area from the JPHC-NEXT (Saku), the Aichi Workers’ Cohort Study (Aichi), the Yuzawa Cohort Study (Yuzawa), and the Yamagata Cohort Study (Yamagata). Those four settings were selected because they all used the JPHC-NEXT FFQ for the exposure assessment for the dietary intake of the entire cohort participants (Figure 1). Study participants were randomly selected from 21,537 individuals who were part of each cohort. The target sample size was calculated according to the method used in the European Prospective Investigation into Cancer and Nutrition (EPIC) Study.^17^^–^^19^

The study was approved by the Institutional Review Boards (IRBs) of Nara Women’s University, Sagami Women’s University, National Cancer Center, Nagoya University, Niigata University. The study protocol was also approved as a part of the Yamagata Cohort Study, in which the entire cohort of the participants of Yamagata city were included instead of random sampling due to the scheduling circumstances, by the IRB of Yamagata University.

### Data collection

To achieve equivalent data collection over the four seasons and days of the week, the designated implementation date and alternative date described in the invitation letter were randomly assigned after the secretariat determined the number of seasons and days of the week by each region. Data on age, height, weight, body mass index (BMI), and physical activity of the participants were collected through the AWARDJP setting. The information on the basic characteristics of the recruited individuals and study population were obtained using a self-reported questionnaire survey collected in each cohort setting prior to this study.^16^

### Dietary assessment using the AWARDJP

The AWARDJP was created with reference to the Automated Multiple-Pass Method (AMPM) developed by the United States Department of Agriculture^20^ as a standard procedure for the 24-hour collection method using a computer. Dietary assessment using AWARDJP mainly consists of six processes (see eTable 1 for details of the multiple-pass approach). This system is equipped with a standardized recipe database for mixed dishes based on the Japanese observational study data.^21^^–^^23^

We adopted two methods (self-administered and interviewer-administered) to collect dietary data through the AWARDJP. Participants were allowed to make the choice of the method in advance depending on if their ability to use a personal computer (PC) at home. Participants who chose the self-administered 24HR could access the system directly from the pre-distributed URL on the specified day using a dedicated ID and password to complete the dietary assessment. It was also possible to save the data once during the meal survey response and continue the response later. For the interviewer-administered method, the interviewers referred to AWARDJP’s standardized manual, asked what the respondents ate and drank in the last 24 hours and their portion size, and immediately entered the information to the AWARDJP. The interviewers were trained according to the standard manual adopted from the International Study of Macro/Micronutrients and Blood Pressure in Japan to avoid interviewer bias.^21^

### Questionnaire on the acceptability of the AWARDJP

After completing the entry for the dietary assessment, the system prompts the participants to answer a survey on the acceptability of the AWARDJP system. In interviewer-administered 24HR, the interviewer asked the participants regarding the questionnaire and entered the answers into the system. This survey included five questions on “meal survey entry time,” “ease of entry,” “ease of food selection,” “availability of dish options,” and “identification of the amount consumed” (see eTable 2 for details of the questionnaire). Regarding the “meal survey entry time,” the interviewer recorded the start and end times for evaluating the total entry time for the interviewer-administered 24HR. The participants self-reported the approximate total entry time in the self-administered 24HR.

### Statistical analysis

The mean and standard deviation (SD) of the participant’s age, height, weight, BMI, and level of physical activity (metabolic equivalent of tasks [METs]·hours/day) were calculated for each cohort. Further, the participants were categorized by each cohort and method, and the distributions and percentages were calculated. As an indicator of AWARDJP acceptability, the median, 25th percentile, and 75th percentile of entry time were calculated for each sex and age group. Mann-Whitney U test was used to evaluate the difference of time required between self-administered 24HR and interviewer-administered 24HR by sex.

Additionally, the proportions of participants who answered each question of acceptability were calculated by age group and response method. The χ^2^ test was performed, and the *P*-value was calculated. Fisher’s exact test (direct method) was used when the number of cells with expected values less than 5 exceeded 20% of the total. The significance level was 5% (two-sided test). SAS 9.4 (SAS Institute Inc., Cary, NC, USA) was used for all data analyses.

## RESULTS

In total, 975 participants from the 5,013 recruited individuals completed the study (response rate: 19.4%), accounting for approximately 4.5% of the total sample of the cohorts. The characteristics of participants who responded to the AWARDJP and those of the total cohort population were similar. These results indicated that the characteristics of the participants represented those of the cohort population (Table 1). Of the 975 participants, 457 (46.9%) and 518 (53.1%) participants chose self-administered and interviewer-administered 24HR, respectively. There were differences in the response method among the regional cohorts (Table 2).

**Table 1.** Characteristics of participants, recruited individuals, and study population

			Saku^a^			Aichi^b^			Yuzawa^c^			Yamagata^d^		
			
			All	Men	Women	All	Men	Women	All	Men	Women	All	Men	Women
Participants^e^ (*n* = 975)			515	226	289	178	137	41	80	37	43	202	71	131
Recruited individuals^f^ (*n* = 5,013)			2,551	1,217	1,342	371	264	107	293	147	146	1,798	580	1,218
Study population^f^ (*n* = 21,537)			11,935	5,528	6,406	3,230	2,397	833	2,554	1,277	1,277	3,818	1,299	2,519

Participation rate^g^			4.3	4.1	4.5	5.5	5.7	4.9	3.1	2.9	3.4	5.3	5.5	5.2

Period	AWARDJP		2017–2018			2016–2017			2016–2017			2017–2018		
	Cohort		2012–2013			2013			2016–2017			2017–2018		

Age, years	Participants^e^	Mean	62.0	62.8	61.4	50.3	50.9	48.2	58.7	59.1	58.3	68.0	68.8	67.5
		SD	9.4	9.5	9.8	5.8	5.8	5.4	9.9	10.2	9.9	7.0	6.7	7.1
	Recruited individuals^f^	Mean	58.1	58.7	57.6	51.4	52.2	49.3	57.9	58.6	57.2	66.2	67.5	65.5
		SD	9.7	9.6	9.8	6.2	6.0	6.2	10.0	9.8	10.1	7.1	6.7	7.2
	Study population^f^	Mean	59.1	58.4	57.9	51.1	51.7	49.5	59.1	59.0	59.2	66.2	67.6	65.6
		SD	9.6	9.6	9.5	6.1	6.0	5.9	9.7	9.6	9.8	6.9	6.5	7.1

Height, cm	Participants^e^	Mean	161.6	168.7	156.1	167.7	170.8	157.3	162.5	168.8	157.1	158.5	166.9	154.0
		SD	8.4	5.6	8.4	8.3	6.3	5.4	8.2	5.7	6.0	8.4	6.9	4.7
	Recruited individuals^f^	Mean	161.7	168.4	155.6	166.4	170.1	157.4	162.4	168.8	155.9	158.3	166.9	154.2
		SD	9.2	7.2	6.1	8.3	6.3	5.2	8.9	6.2	6.1	8.1	6.0	5.3
	Study population^f^	Mean	161.7	168.6	155.7	167.1	170.2	157.9	162.0	168.4	155.5	158.5	166.7	154.3
		SD	8.8	6.4	5.7	7.7	5.6	5.3	8.9	6.4	5.9	8.1	5.9	5.4

Weight, kg	Participants^e^	Mean	59.9	66.1	55.0	64.7	68.0	53.7	60.6	66.6	55.4	57.5	66.6	52.6
		SD	10.7	9.8	8.2	10.5	9.4	5.7	11.1	10.9	8.7	11.0	10.8	7.4
	Recruited individuals^f^	Mean	60.6	67.2	54.7	64.0	68.1	53.6	61.3	68.1	54.4	57.6	66.1	53.6
		SD	11.2	9.5	9.0	11.2	9.7	7.7	13.0	12.3	9.7	10.5	9.7	8.2
	Study population^f^	Mean	60.6	67.4	54.7	64.5	68.1	54.0	60.5	67.2	53.8	57.9	65.9	53.7
		SD	11.2	9.7	8.8	11.1	9.5	8.1	12.2	11.1	9.2	10.4	9.5	8.2

BMI, kg/m^2^	Participants^e^	Mean	22.8	23.2	22.5	23.0	23.3	21.8	22.8	23.3	22.4	22.8	23.8	22.2
		SD	2.9	2.8	3.0	2.8	2.8	2.5	3.3	3.5	3.1	2.9	2.9	2.8
	Recruited individuals^f^	Mean	23.2	23.8	22.7	23.0	23.5	21.7	23.1	23.8	22.4	22.9	23.7	22.5
		SD	5.6	5.6	5.6	3.2	3.2	2.8	3.7	3.7	3.6	3.2	2.9	3.2
	Study population^f^	Mean	23.1	23.7	22.6	23.0	23.5	21.6	22.9	23.6	22.2	22.9	23.7	22.6
		SD	3.9	3.7	4.0	3.2	3.1	3.1	3.6	3.3	3.7	3.2	2.9	3.2

Physical activity level, METs·hours/day	Participants^e^	Mean	39.9	39.8	40.0	36.6	36.5	37.0	39.9	41.5	38.6	36.6	36.7	36.6
		SD	5.8	6.1	5.6	3.0	3.1	2.9	6.9	8.2	5.3	5.4	6.2	5.0
	Recruited individuals^f^	Mean	40.8	41.2	40.4	36.9	36.4	38.1	40.6	40.8	40.4	36.7	36.5	36.7
		SD	6.6	7.2	5.9	3.5	2.8	4.5	6.7	6.7	6.6	5.7	6.3	5.5
	Study population^f^	Mean	40.6	41.1	40.2	36.8	36.5	37.9	41.0	41.5	40.6	36.8	37.1	36.7
		SD	6.4	7.0	5.8	3.4	3.0	4.2	6.9	7.4	6.3	5.7	6.9	5.1

BMI, body mass index; JPHC-NEXT, Japan Public Health Center-based Prospective Study for the Next Generation; mean, arithmetic mean; METs, metabolic equivalent of tasks; SD, standard deviation.

^a^Saku area in the JPHC-NEXT, ^b^Aichi Workers’ Cohort Study, ^c^Yuzawa Cohort Study, ^d^Yamagata Cohort Study.

^e^Participants are categorized using data obtained from AWARDJP.

^f^Study population are collected from part of each cohort. Recruited individuals and study population are categorized using data obtained from the JPHC-NEXT survey.

^g^Percentage of participants over the study population.

**Table 2.** Response status of AWARDJP respondents

	Total						Saku^a^						Aichi^b^						Yuzawa^c^						Yamagata^d^					
				
	**Total**		Self- administered^h^		Interviewer- administered^i^		**Total**		Self- administered^h^		Interviewer- administered^i^		**Total**		Self- administered^h^		Interviewer- administered^i^		**Total**		Self- administered^h^		Interviewer- administered^i^		**Total**		Self- administered^h^		Interviewer- administered^i^	
				
	***n***	**%**	*n*	%	*n*	%	***n***	**%**	*n*	%	*n*	%	***n***	**%**	*n*	%	*n*	%	***n***	**%**	*n*	%	*n*	%	***n***	**%**	*n*	%	*n*	%
Total number of participants	**975**		457	46.9	518	53.1	**515**		220	42.7	295	57.3	**178**		168	94.4	10	5.6	**80**		32	40.0	48	60.0	**202**		37	18.3	165	81.7

Sex																														
Men	**471**	**48.3**	292	63.9	179	34.6	**226**	**43.9**	123	55.9	103	34.9	**137**	**77.0**	129	76.8	8	80.0	**37**	**46.3**	22	68.8	15	31.3	**71**	**35.1**	18	48.6	53	32.1
Women	**504**	**51.7**	165	36.1	339	65.4	**289**	**56.1**	97	44.1	192	65.1	**41**	**23.0**	39	23.2	2	20.0	**43**	**53.8**	10	31.3	33	68.8	**131**	**64.9**	19	51.4	112	67.9

Age group																														
<50 years	**182**	**18.7**	148	32.4	34	6.6	**74**	**14.4**	53	24.1	21	7.1	**84**	**47.2**	80	47.6	4	40.0	**19**	**23.8**	11	34.4	8	16.7	**5**	**2.5**	4	10.8	1	0.6
50–59 years	**262**	**26.9**	183	40.0	79	15.3	**132**	**25.6**	83	37.7	49	16.6	**83**	**46.6**	79	47.0	4	40.0	**22**	**27.5**	11	34.4	11	22.9	**25**	**12.4**	10	27.0	15	9.1
60–69 years	**269**	**27.6**	92	20.1	177	34.2	**178**	**34.6**	64	29.1	114	38.6	**11**	**6.2**	9	5.4	2	20.0	**27**	**33.8**	7	21.9	20	41.7	**53**	**26.2**	12	32.4	41	24.8
≥70 years	**262**	**26.9**	34	7.4	228	44.0	**131**	**25.4**	20	9.1	111	37.6	**0**	**0**	0	0	0	0	**12**	**15.0**	3	9.4	9	18.8	**119**	**58.9**	11	29.7	108	65.5

People who required reminders																														
0 times^e^	**614**	**63.0**	272	59.5	342	66.0	**350**	**68.0**	143	65.0	207	70.2	**87**	**48.9**	81	48.2	6	60.0	**43**	**53.8**	22	68.8	21	43.8	**134**	**66.3**	26	70.3	108	65.5
Once	**240**	**24.6**	126	27.6	114	22.0	**114**	**22.1**	51	23.2	63	21.4	**60**	**33.7**	56	33.3	4	40.0	**17**	**21.3**	10	31.3	7	14.6	**49**	**24.3**	9	24.3	40	24.2
Twice	**121**	**12.4**	59	12.9	62	12.0	**51**	**9.9**	26	11.8	25	8.5	**31**	**17.4**	31	18.5	0	0	**20**	**25.0**	0	0	20	41.7	**19**	**9.4**	2	5.4	17	10.3

Assessment days																														
1 day only	**379**	**38.9**	300	65.6	79	15.3	**200**	**38.8**	153	69.5	47	15.9	**100**	**56.2**	100	59.5	0	0	**31**	**38.8**	22	68.8	9	18.8	**48**	**23.8**	25	67.6	23	13.9
2 days	**572**	**58.7**	143	31.3	429	82.8	**299**	**58.1**	58	26.4	241	81.7	**74**	**41.6**	65	38.7	9	90	**48**	**60.0**	9	28.1	39	81.3	**151**	**74.8**	11	29.7	140	84.8
3 days or more	**24**	**2.5**	14	3.1	10	1.9	**16**	**3.1**	9	4.1	7	2.4	**4**	**2.2**	3	1.8	1	10	**1**	**1.3**	1	3.1	0	0	**3**	**1.5**	1	2.7	2	1.2

Number of participants on the requested day of the week^f^																														
Total	**650**	**66.7**	270	59.1	380	73.4	**350**	**68.0**	132	60.0	218	73.9	**103**	**57.9**	93	55.4	10	100	**54**	**67.5**	23	71.9	31	64.6	**143**	**70.8**	22	59.5	121	73.3
Monday	**99**	**68.3**	42	62.7	57	73.1	**54**	**73.0**	17	65.4	37	77.1	**23**	**67.6**	19	63.3	4	100	**7**	**77.8**	3	75.0	4	80.0	**15**	**53.6**	3	42.9	12	57.1
Tuesday	**93**	**63.3**	39	61.9	54	64.3	**50**	**62.5**	20	60.6	30	63.8	**13**	**56.5**	12	54.5	1	100	**13**	**86.7**	4	100	9	81.8	**17**	**58.6**	3	75.0	14	56.0
Wednesday	**96**	**65.8**	39	59.1	57	71.3	**53**	**65.4**	20	58.8	33	70.2	**14**	**63.6**	12	60.0	2	100	**4**	**50.0**	3	50.0	1	50.0	**25**	**71.4**	4	66.7	21	72.4
Thursday	**106**	**71.6**	41	69.5	65	73.0	**54**	**71.1**	20	64.5	34	75.6	**9**	**60.0**	9	60.0	0		**14**	**77.8**	8	100	6	60.0	**29**	**74.4**	4	80	25	73.5
Friday	**85**	**65.9**	27	54.0	58	73.4	**51**	**71.8**	17	65.4	34	75.6	**8**	**44.4**	7	41.2	1	100	**4**	**36.4**	1	33.3	3	37.5	**22**	**75.9**	2	50.0	20	80.0
Saturday	**85**	**60.3**	38	47.5	47	77.0	**41**	**55.4**	17	43.6	24	68.6	**16**	**53.3**	16	53.3	0		**7**	**58.3**	2	40.0	5	71.4	**21**	**84.0**	3	50.0	18	94.7
Sunday	**86**	**72.3**	44	61.1	42	89.4	**47**	**79.7**	21	67.7	26	92.9	**20**	**55.6**	18	52.9	2	100	**5**	**71.4**	2	100	3	60.0	**14**	**82.4**	3	60.0	11	91.7

Response day																														
Weekdays (Monday–Friday)	**715**	**73.3**	305	66.7	410	79.2	**382**	**74.2**	150	68.2	232	78.6	**112**	**62.9**	104	61.9	8	80.0	**61**	**76.3**	25	78.1	36	75.0	**160**	**79.2**	26	70.3	134	81.2
Weekend (Saturday, Sunday)	**260**	**26.7**	152	33.3	108	20.8	**133**	**25.8**	70	31.8	63	21.4	**66**	**37.1**	64	38.1	2	20.0	**19**	**23.8**	7	21.9	12	25.0	**42**	**20.8**	11	29.7	31	18.8
Monday	**145**	**14.9**	67	14.7	78	15.1	**74**	**14.4**	26	11.8	48	16.3	**34**	**19.1**	30	17.9	4	40.0	**9**	**11.3**	4	12.5	5	10.4	**28**	**13.9**	7	18.9	21	12.7
Tuesday	**147**	**15.1**	63	13.8	84	16.2	**80**	**15.5**	33	15.0	47	15.9	**23**	**12.9**	22	13.1	1	10.0	**15**	**18.8**	4	12.5	11	22.9	**29**	**14.4**	4	10.8	25	15.2
Wednesday	**146**	**15.0**	66	14.4	80	15.4	**81**	**15.7**	34	15.5	47	15.9	**22**	**12.4**	20	11.9	2	20.0	**8**	**10.0**	6	18.8	2	4.2	**35**	**17.3**	6	16.2	29	17.6
Thursday	**148**	**15.2**	59	12.9	89	17.2	**76**	**14.8**	31	14.1	45	15.3	**15**	**8.4**	15	8.9	0	0	**18**	**22.5**	8	25.0	10	20.8	**39**	**19.3**	5	13.5	34	20.6
Friday	**129**	**13.2**	50	10.9	79	15.3	**71**	**13.8**	26	11.8	45	15.3	**18**	**10.1**	17	10.1	1	10.0	**11**	**13.8**	3	9.4	8	16.7	**29**	**14.4**	4	10.8	25	15.2
Saturday	**141**	**14.5**	80	17.5	61	11.8	**74**	**14.4**	39	17.7	35	11.9	**30**	**16.9**	30	17.9	0	0	**12**	**15.0**	5	15.6	7	14.6	**25**	**12.4**	6	16.2	19	11.5
Sunday	**119**	**12.2**	72	15.8	47	9.1	**59**	**11.5**	31	14.1	28	9.5	**36**	**20.2**	34	20.2	2	20.0	**7**	**8.8**	2	6.3	5	10.4	**17**	**8.4**	5	13.5	12	7.3

Response date season^g^																														
Spring	**174**	**17.8**	68	14.9	106	20.5	**134**	**26.0**	58	26.4	76	25.8	**3**	**1.7**	3	1.8	0	0	**2**	**2.5**	0	0	2	4.2	**35**	**17.3**	7	18.9	28	17.0
Summer	**232**	**23.8**	103	22.5	129	24.9	**123**	**23.9**	57	25.9	66	22.4	**27**	**15.2**	27	16.1	0	0	**17**	**21**	9	28.1	8	16.7	**65**	**32.2**	10	27.0	55	33.3
Autumn	**281**	**28.8**	142	31.1	139	26.8	**137**	**26.6**	62	28.2	75	25.4	**61**	**34.3**	57	33.9	4	40.0	**23**	**28.8**	11	34.4	12	25.0	**60**	**29.7**	12	32.4	48	29.1
Winter	**288**	**29.5**	144	31.5	144	27.8	**121**	**23.5**	43	19.5	78	26.4	**87**	**48.9**	81	48.2	6	60.0	**38**	**47.5**	12	37.5	26	54.2	**42**	**20.8**	8	21.6	34	20.6

Number of days from survey request to response																														
Median	**17**		18		17		**17**		18		16		**41**		43		18		**17**		8		49		**16**		13		17	
(Q1, Q3)^j^	**(6,**	**70)**	(5,	64)	(7,	76)	**(3,**	**73)**	(6.5,	77)	(8,	69)	**(4,**	**58)**	(5,	59)	(4,	47)	**(3,**	**69)**	(1,	30)	(5.5,	80)	**(5,**	**75)**	(2,	64)	(6,	77)

AWARDJP, Automated Web-based assessment System Using Recipe-Data for Japanese; JPHC-NEXT, Japan Public Health Center-based Prospective Study for the Next Generation.

The percentage of “total” shown in bold is the percentage of the total number of participants in each cohort. Percentages by response method (self-administered/interviewer-administered) are expressed as percentages of the total number of participants who used each method.

^a^Saku area in the JPHC-NEXT, ^b^Aichi Workers’ Cohort Study, ^c^Yuzawa Cohort Study, ^d^Yamagata Cohort Study.

^e^Reminders are set to 0 times for those who responded to the initial survey request.

^f^Participants are given a choice to select one out of two days within a week to take the assessment.

Percentage of respondents on the requested day is calculated as follows: “number of participants who took the survey according to our request”/“total number of participants” × 100.

For those who responded to our request for two days, the dietary survey on the first day was considered for the calculation.

^g^The seasons are set as follows. Spring from March to May, summer from June to August, autumn from September to November, and winter from December to February. Those who responded for 2 days or more were shown the season when they responded to the dietary survey on the first day.

^h^Self-administered web-based dietary 24-hour recall.

^i^Interviewer-administered telephone-based 24-hour recall.

^j^Q1: 1st quintile; Q3: 3rd quintile.

**Figure 2.**
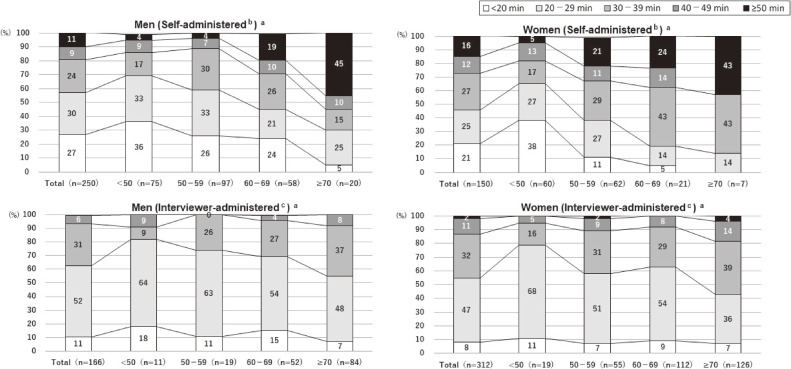
Questionnaire on AWARDJP acceptability: distribution of response time required for the self-administered and interviewer-administered 24-hour dietary recall. AWARDJP, Automated Web-based assessment System Using Recipe-Data for Japanese. Missing values are excluded for each item. ^a^Distribution of response time required for self-administered web-based dietary 24-hour recall and interviewer-administered telephone-based 24-hour recall are shown by sex and age group. ^b^Self-administered web-based dietary 24-hour recall. ^c^Interviewer-administered telephone-based 24-hour recall.

Table 3 presents the duration of the dietary survey by method, sex, and age. Among men, the median time required to complete the dietary survey was 20 (interquartile range [IQR], 15–30) and 25 (IQR, 21–31) minutes in the self-administered and interviewer-administered 24HR, respectively. For women, it was 30 (IQR, 20–40) and 28 (IQR, 23–35) minutes, respectively. Furthermore, when aggregated by age, the response time of the self-administered 24HR tended to be longer than that of the interviewer-administered 24HR as the participant age increased. We found a tendency of a broader range of variation by age in self-administered 24HR than interview-administered 24HR. In the case of self-administered 24HR, there were participants whose assessment time >50 minutes for all age groups and sexes. For interviewer-administered 24HR, an assessment time >50 minutes was rare across all age groups (Figure 2).

**Table 3.** Questionnaire on AWARDJP acceptability: comparison of the time required (minutes) for both the self-administered and interviewer-administered 24-hour recall

	Men							Women						

	*n*	25th percentile	Median	75th percentile	Min	Max	*P*-value^c^	*n*	25th percentile	Median	75th percentile	Min	Max	*P*-value^c^
Total														
Self-administered^a^	250	15	20	30	3	120	0.008	150	20	30	40	5	209	0.364
Interviewer-administered^b^	166	21	25	31	10	48		312	23	28	35	12	60	

<50 years														
Self-administered^a^	75	15	20	30	3	60	0.134	60	15	20	30	10	60	0.112
Interviewer-administered^b^	11	20	23	28	16	40		19	21	24	28	15	45	

50–59 years														
Self-administered^a^	97	15	20	30	10	60	0.416	62	20	30	40	5	209	0.385
Interviewer-administered^b^	19	21	25	30	10	35		55	23	27	33	14	50	

60–69 years														
Self-administered^a^	58	20	30	40	5	100	0.341	21	30	30	40	10	120	0.005
Interviewer-administered^b^	52	20	24	30	12	40		112	23	27	30	12	60	

≥70 years														
Self-administered^a^	20	20	40	60	10	120	0.023	7	30	30	60	20	90	0.157
Interviewer-administered^b^	84	22	28	35	14	48		126	25	30	35	13	60	

AWARDJP, Automated Web-based assessment System Using Recipe-Data for Japanese.

Missing values are excluded for each item.

^a^Self-administered web-based dietary 24-hour recall.

^b^Interviewer-administered telephone-based 24-hour recall.

^c^Mann-Whitney U test.

Table 4A and Table 4B shows responses of the respondents regarding the acceptability of the AWARDJP. More than 60% in the self-administered 24HR and approximately 10% in the interviewer-administered 24HR answered either “somewhat difficult” or “difficult” regarding “ease of entry.” For the self-administered 24HR, the percentage of those who answered “somewhat difficult” or “difficult” showed an increasing trend with age for both men and women. Regarding the question about the difficult part to enter, the most common answer among those in the self-administered 24HR was “dish name selection” (about 70%), followed by “entering cooking details” (about 60%). However, there was almost no significant difference by sex (eTable 3A and eTable 3B).

**Table 4A.** Questionnaire on AWARDJP acceptability: comparison by response method and age in men (*n* = 471)

	Total					<50 years					50–59 years					60–69 years					≥70 years				
				
	Self-administered^a^ (*n* = 292)		Interviewer- administered^b^ (*n* = 179)		*P* value^c^	Self-administered^a^ (*n* = 86)		Interviewer- administered^b^ (*n* = 12)		*P* value^c^	Self- administered^a^ (*n* = 112)		Interviewer- administered^b^ (*n* = 19)		*P* value^c^	Self- administered^a^ (*n* = 67)		Interviewer- administered^b^ (*n* = 56)		*P* value^c^	Self- administered^a^ (*n* = 27)		Interviewer- administered^b^ (*n* = 92)		*P* value^c^
									
	*n*	%	*n*	%		*n*	%	*n*	%		*n*	%	*n*	%		*n*	%	*n*	%		*n*	%	*n*	%	
Ease of entry																									
Easy	27	10.8	103	62.4	<0.001	11	14.7	8	72.7	0.001	10	10.4	13	68.4	<0.001	6	10.3	39	76.5	<0.001	0	0.0	43	51.2	<0.001
Relatively easy	60	24.1	41	24.9		25	33.3	2	18.2		25	26.0	4	21.1		10	17.2	7	13.7		0	0.0	28	33.3	
Somewhat difficult	124	49.8	16	9.7		33	44.0	1	9.1		50	52.1	2	10.5		31	54	5	9.8		10	50.0	8	9.5	
Difficult	38	15.3	5	3.0		6	8.0	0	0.0		11	11.5	0	0.0		11	19.0	0	0.0		10	50.0	5	6.0	

Difficult part to enter^d^																									
Log in and select a date	11	6.8	N/A			3	7.7	N/A			1	1.6	N/A			3	7.1	N/A			4	20.0	N/A		
Meal time and occasion	17	10.5	0	0.0	0.226	8	20.5	0	0.0	1.000	3	4.9	0	0.0	1.000	2	4.8	0	0.0	1.000	4	20.0	0	0.0	0.136
Dish name selection	116	71.6	9	42.9	0.008	28	71.8	1	100	1.000	44	72.1	0	0.0	0.088	34	81.0	3	60.0	0.285	10	50.0	5	38.5	0.515
Entering cooking details/telling cooking details^f^	91	56.2	7	33.3	0.048	17	43.6	1	100	0.450	40	65.6	1	50.0	1.000	24	57.1	2	40.0	0.644	10	50.0	3	23.1	0.122
Final confirmation screen	10	6.2	0	0.0	0.608	1	2.6	0	0.0	1.000	4	6.6	0	0.0	1.000	3	7.1	0	0.0	1.000	2	10.0	0	0.0	0.508
Others	12	7.4	5	23.8	0.030	3	7.7	0	0.0	1.000	5	8.2	0	0.0	1.000	2	4.8	2	40.0	0.051	2	10.0	3	23.1	0.360

Ease of food selection																									
Easy/agree^f^	30	22.1	120	80.5	<0.001	14	31.8	7	70.0	0.162	11	18.0	16	94.1	<0.001	5	19.2	39	84.8	<0.001	0	0.0	58	76.3	<0.001
Relatively easy/somewhat agree^f^	57	41.9	27	18.1		19	43.2	3	30.0		28	45.9	1	5.9		9	34.6	7	15.2		1	20.0	16	21.1	
Somewhat difficult/somewhat disagree^f^	40	29.4	1	0.7		8	18.2	0	0.0		18	29.5	0	0.0		11	42.3	0	0.0		3	60.0	1	1.3	
Difficult/disagree^f^	9	6.6	1	0.7		3	6.8	0	0.0		4	6.6	0	0.0		1	3.9	0	0.0		1	20.0	1	1.3	

Availability of dish options																									
Agree	47	35.1	—			19	43.2	—			21	35.0	—			7	26.9	—			0	0.0	—		
Somewhat agree	59	44.0	—			18	40.9	—			29	48.3	—			10	38.5	—			2	50.0	—		
Somewhat disagree	22	16.4	—			6	13.6	—			8	13.3	—			7	26.9	—			1	25.0	—		
Disagree	6	4.5	—			1	2.3	—			2	3.3	—			2	7.7	—			1	25.0	—		

Alternative options when dishes were not available^e^																									
I was able to add it myself as a new dish	0	0.0	—			0	0.0	—			0	0.0	—			0	0.0	—			0	0.0	—		
I chose a similar dish	10	35.7	—			0	0.0	—			3	30.0	—			5	55.6	—			2	100	—		
I added/deleted foods of similar recipes	2	7.1	—			0	0.0	—			1	10.0	—			1	11.1	—			0	0.0	—		
I did not make an entry	1	3.6	—			0	0.0	—			0	0.0	—			1	11.1	—			0	0.0	—		
Others	0	0.0	—			0	0.0	—			0	0.0	—			0	0.0	—			0	0.0	—		

Identification of the amount you consumed																									
Agree	57	48.3	128	78.5	<0.001	20	47.6	8	72.7	0.552	26	51.0	14	73.7	0.155	8	38.1	40	80.0	0.000	3	75.0	66	79.5	0.186
Somewhat agree	44	37.3	31	19.0		17	40.5	3	27.3		19	37.3	5	26.3		8	38.1	9	18.0		0	0.0	14	16.9	
Somewhat disagree	16	13.6	3	1.8		4	9.5	0	0.0		6	11.8	0	0.0		5	23.8	0	0.0		1	25.0	3	3.6	
Disagree	1	0.8	1	0.6		1	2.4	0	0.0		0	0.0	0	0.0		0	0.0	1	2.0		0	0.0	0	0.0	

AWARDJP, Automated Web-based assessment System Using Recipe-Data for Japanese; N/A, not applicable.

The “total” percentage is expressed as a percentage of the total number of participants. The percentage of self-administered/interviewer-administered responses is expressed as the percentage of the total number of those who used each method.

Missing values are excluded for each item.

—: No description because the question was answered by the interviewer.

^a^Self-administered web-based dietary 24-hour recall.

^b^Interviewer-administered telephone-based 24-hour recall.

^c^χ^2^ test: Fisher’s exact test (direct method) was used when the number of cells with expected values less than 5 exceeded 20% of the total.

^d^The denominator is the number of subjects who selected “Somewhat difficult” or “Difficult” in the above question.

^e^The denominator is the number of subjects who selected “Somewhat disagree” or “Disagree” in the above question.

^f^Response method: Self-administered/Interviewer-administered.

**Table 4B.** Questionnaire on AWARDJP acceptability: comparison by response method and age group in women (*n* = 504)

	Total					<50 years					50–59 years					60–69 years					≥70 years				
				
	Self- administered^a^ (*n* = 165)		Interviewer- administered^b^ (*n* = 339)		*P* value^c^	Self- administered^a^ (*n* = 62)		Interviewer- administered^b^ (*n* = 22)		*P* value^c^	Self- administered^a^ (*n* = 71)		Interviewer- administered^b^ (*n* = 60)		*P* value^c^	Self- administered^a^ (*n* = 25)		Interviewer- administered^b^ (*n* = 121)		*P* value^c^	Self- administered^a^ (*n* = 7)		Interviewer- administered^b^ (*n* = 136)		*P* value^c^
									
	*n*	%	*n*	%		*n*	%	*n*	%		*n*	%	*n*	%		*n*	%	*n*	%		*n*	%	*n*	%	
Ease of entry																									
Easy	13	8.6	210	67.1	<0.001	7	11.7	14	70.0	<0.001	6	9.7	42	76.4	<0.001	0	0.0	75	67.0	<0.001	0	0.0	79	62.7	<0.001
Relatively easy	35	23.2	77	24.6		21	35.0	5	25.0		9	14.5	9	16.4		5	22.7	27	24.1		0	0.0	36	28.6	
Somewhat difficult	76	50.3	23	7.3		24	40.0	1	5.0		38	61.3	4	7.3		11	50.0	8	7.1		3	42.9	10	7.9	
Difficult	27	17.9	3	1.0		8	13.3	0	0.0		9	14.5	0	0.0		6	27.3	2	1.8		4	57.1	1	0.8	

Difficult part to enter^d^																									
Log in and select a date	6	5.8	N/A			2	6.3	N/A			4	8.5	N/A			0	0.0	N/A			0	0.0	N/A		
Meal time and occasion	13	12.6	1	3.8	0.299	5	15.6	0	0.0	1.000	4	8.5	0	0.0	1.000	2	11.8	1	10.0	1.000	2	28.6	0	0.0	0.137
Dish name selection	74	71.8	4	15.4	<0.001	18	56.3	0	0.0	0.455	38	80.9	1	25.0	0.036	14	82.4	1	10.0	0.001	4	57.1	2	18.2	0.141
Entering cooking details/telling cooking details^f^	71	68.9	12	46.2	0.030	23	71.9	1	100	1.000	31	66.0	2	50.0	0.607	12	70.6	3	30.0	0.057	5	71.4	6	54.5	0.637
Final confirmation screen	9	8.7	0	0.0	0.203	3	9.4	0	0.0	1.000	2	4.3	0	0.0	1.000	1	5.9	0	0.0	1.000	3	42.9	0	0.0	0.043
Others	6	5.8	7	26.9	0.005	1	3.1	0	0.0	1.000	2	4.3	1	25.0	0.221	1	5.9	4	40.0	0.047	2	28.6	2	18.2	1.000

Ease of food selection																									
Easy/agree^f^	15	17.6	234	78.8	<0.001	6	20.7	16	84.2	<0.001	8	19.5	43	82.7	<0.001	1	7.7	79	75.2	<0.001	0	0.0	96	79.3	0.001
Relatively easy/somewhat agree^f^	37	43.5	60	20.2		12	41.4	3	15.8		18	43.9	9	17.3		7	53.8	26	24.8		0	0.0	22	18.2	
Somewhat difficult/somewhat disagree^f^	29	34.1	3	1.0		9	31.0	0	0.0		15	36.6	0	0.0		4	30.8	0	0.0		1	50.0	3	2.5	
Difficult/disagree^f^	4	4.7	0	0.0		2	6.9	0	0.0		0	0.0	0	0.0		1	7.7	0	0.0		1	50.0	0	0.0	

Availability of dish options																									
Agree	27	31.8	—			13	40.6	—			11	28.2	—			3	25.0	—			0	0.0	—		
Somewhat agree	34	40.0	—			12	37.5	—			14	35.9	—			7	58.3	—			1	50.0	—		
Somewhat disagree	23	27.1	—			6	18.8	—			14	35.9	—			2	16.7	—			1	50.0	—		
Disagree	1	1.2	—			1	3.1	—			0	0.0	—			0	0.0	—			0	0.0	—		

Alternative options when dishes were not available^e^																									
I was able to add it myself as a new dish	0	0.0	—			0	0.0	—			0	0.0	—			0	0.0	—			0	0.0	—		
I chose a similar dish	10	41.7	—			2	28.6	—			6	42.9	—			2	100	—			0	0.0	—		
I added/deleted foods of similar recipes	4	16.7	—			1	14.3	—			2	14.3	—			0	0.0	—			1	100	—		
I did not make an entry	0	0.0	—			0	0.0	—			0	0.0	—			0	0.0	—			0	0.0	—		
Others	0	0.0	—			0	0.0	—			0	0.0	—			0	0.0	—			0	0.0	—		

Identification of the amount you consumed																									
Agree	45	55.6	249	80.1	<0.001	20	66.7	18	90.0	0.178	20	54.1	47	85.5	0.003	5	41.7	85	77.3	0.007	0	0.0	99	78.6	0.022
Somewhat agree	23	28.4	52	16.7		7	23.3	2	10.0		10	27.0	6	10.9		5	41.7	23	20.9		1	50.0	21	16.7	
Somewhat disagree	13	16.0	9	2.9		3	10.0	0	0.0		7	18.9	2	3.6		2	16.7	1	0.9		1	50.0	6	4.8	
Disagree	0	0.0	1	0.3		0	0.0	0	0.0		0	0.0	0	0.0		0	0.0	1	0.9		0	0.0	0	0.0	

AWARDJP, Automated Web-based assessment System Using Recipe-Data for Japanese; N/A, not applicable.

The “total” percentage is expressed as a percentage of the total number of participants. The percentage of self-administered/interviewer-administered responses is expressed as the percentage of the total number of those who used each method.

Missing values are excluded for each item.

—: No description because the question was answered by the interviewer.

^a^Self-administered web-based dietary 24-hour recall.

^b^Interviewer-administered telephone-based 24-hour recall.

^c^χ^2^ test: Fisher’s exact test (direct method) was used when the number of cells with expected values less than 5 exceeded 20% of the total.

^d^The denominator is the number of subjects who selected “Somewhat difficult” or “Difficult” in the above question.

^e^The denominator is the number of subjects who selected “Somewhat disagree” or “Disagree” in the above question.

^f^Response method: Self-administered/Interviewer-administered.

## DISCUSSION

To evaluate the applicability of the AWARDJP in large-scale epidemiological studies, this study was conducted with two aims: to examine the sampling rate and respondent characteristics of the participants randomly sampled from the four selected study cohorts, and to clarify the difference in the acceptability among the participants by the assessment method. Referring to the EPIC study, where the random sample size was 5–12% of the cohort population for FFQ calibration, this study included only 4.5% of the total cohort sample. However, the characteristics of the participants and those of the study population were quite consistent, indicating the possibility of AWARDJP applicability to large-scale epidemiological studies for 24HR dietary assessment and for FFQ calibration. To use it for correcting the measurement error in FFQs, further studies considering the acceptance rate obtained in this study are required.

The median time required to enter responses into the system was 20 and 25 minutes for men, and 30 and 28 minutes for women for the self-administered and interviewer-administered 24HR, respectively. These results were consistent with those of the review study conducted by Eldrige^24^ on the evaluation of dietary assessment tools, where it was observed that the average time to complete an assessment ranged from at least 14 minutes to 45–60 minutes. This indicated a similarity with the AWARDJP system, from a data entry time perspective. However, there were several differences between the interviewer-administered 24HR and the self-administered 24HR. In this study, especially in the case of self-administered 24HR survey, it is not possible to accurately confirm whether the participant was interrupted in the middle or ended the survey prematurely, which is a limitation of this study.

Additionally, the proportion of those who answered “somewhat difficult” or “difficult” to the question regarding ease of system use was about 10% in the interviewer-administered 24HR group, while it was over 60% in the self-administered 24HR. In the interviewer-administered 24HR, the interviewer entered the data for the participants, shortening the data entry time, possibly avoiding some of the difficult processes affecting the acceptability of the system. Based on the above, the evaluation of AWARDJP system acceptability may have been biased by age group or sex.

The dish name selection and the entering cooking details (food and food volume) were the most difficult points to answer according to the result of the acceptability survey. A better approach for entering cooking details, such as the dish name selection option and search keywords, may be necessary to improve the system so that the number of web-responders would increase.

The result of the questionnaire on AWARDJP acceptability by the web-based self-administered 24HR showed that many respondents answered that it was difficult to select a dish name from the food search interface and enter the cooking details into the system. To use the web-based 24HR for large-scale epidemiological studies, it is necessary to improve the food search interface for ease of use and support methods, considering that the web environment is being used by users who are not accustomed to PCs. Furthermore, it will be necessary to examine the accuracy and validity of both the response methods based on accurate indicators, such as a biomarker (eg, double-labeled water method, 24-hour urine storage method).^11^^–^^13^^,^^25^

## SUPPLEMENTARY MATERIAL

eTable 1. Application of the automated multiple-pass method (AMPM) for the AWARDJP

eTable 2. Questionnaire on AWARDJP acceptability

eTable 3A. Questionnaire on AWARDJP acceptability: comparison by sex and age group in self-administered 24HR^a^ (n = 457)

eTable 3B. Questionnaire on AWARDJP acceptability: comparison by sex and age group in interviewer-administered 24HR^b^ (n = 518)
