# Supplementary material for: Applicability of a Web-based 24-hour Dietary Recall Tool for Japanese Populations in Large-scale Epidemiological Studies
Source: J Epidemiol. 2023 Aug 5;33(8):419–27. doi: 10.2188/jea.JE20220071 (PMC10319522; doi:10.2188/jea.JE20220071)
Supplement: Supplementary file 1 [file je-33-419-s001.pdf]

eTable 1. Application of the automated multiple-pass method (AMPM) for the AWARDJP

AWARDJP	
Step	Purpose
(1) Log in and select a date	The participant logins into the system with a survey ID and password, followed by selecting the date of the meal consume.
(2) Mealtime and occasion (Corresponding to time and occasion in AMPM)	Select scene(s) of the meal taken, which can be breakfast, lunch, dinner, and (or) snacks. A question regarding the meal scene is prompted to answer about mealtime, place, and people who were eating with.
(3) Dish name selection (Corresponding to quicklist in AMPM)	Search for meal names taken from a list of major categories or enter a search keyword. Dishes that correspond to the search contents are displayed from the dish database, and the respondent makes entries for each meal taken. The AWARDJP carries meal names composed of traditional dish names and name of main ingredients (for example, miso soup with tofu and seaweed). Corresponding meal names can be searched by entering the name of the dish or the main ingredients as keywords. If the dish name is not on the list, respondents can choose a similar dish. In the interviewer-administered 24HR, the investigator explains to the respondent that the dish name is not on the list and reflects the respondent's intentions to choose a similar dish.
(4) Entering cooking details (Corresponding to detail cycle in AMPM)	Enter the amount for each food and its details regarding the food composition. First, select the portion size (selectable portion sizes are 0.1 to 5.0 times with 0.1 times increments). A list of standard food weights per portion size selected is displayed on the next screen. Here, it is possible to edit the food composition and change the weight of each food by adding or deleting foods (including seasonings for seasoning). Also, on the next screen, a question about the presence or absence of additional seasonings such as table soy sauce will be displayed. If added, select the seasoning, and enter the amount used (select the number of tablespoons or teaspoons).
(5) Final confirmation screen (Corresponding to forgotten food list and final probe in AMPM)	Dishes entered are listed in chronological order and by meal. If corrections need to be made, the respondent can return to the edit screen and make corrections to edit the details of the meal. Finally, check whether you are eating or drinking food that makes it easy to forget to enter drinks and sweets.
(6) Other	Habitual intake confirmation (whether the amount of meal consumed was more than usual or less than usual)

24HR, 24-hour dietary recall; AMPM, Automated Multiple-Pass Method, developed by the United States Department of Agriculture.

AWARDJP, the Automated Web-based assessment System Using Recipe-Data for Japanese.

eTable 2. Questionnaire on AWARDJP acceptability

	Self-administered [a]	Interviewer-administered [b]
**Ease of entry** [c]	How did you feel about entering your meals using this system?	How did you feel about the telephone-base assessment?
**Difficult part to enter** [d]	Answer only if you selected "Somewhat difficult" or "Difficult" in the above question:	Answer only if you selected "Somewhat difficult" or "Difficult" in the above question:
	What did you find particularly difficult? Please select all from the following items.	What did you find particularly difficult to tell the investigators? Please select all from the following items.
	Log in and select a date	–
	Meal time and occasion	Dish name selection
	Dish name selection	Telling cooking details
	Entering cooking details	Final confirmation screen
	Final confirmation screen	Others
	Others	
**Ease of food selection** [c]	How did you feel about the food choices you made when entering your meal?	Did you tell the telephone investigator exactly what kind of food you ate?
**Availability of dish options** [c]	Was the dish you wanted to enter mostly included in the options?	Was the dish respondent wanted to enter mostly included in the options? (Interviewer answered)
**Alternative options when dishes were not available** [c]	Answer only if you selected "somewhat disagree" or "disagree" in the above question:	Answer only if you selected "somewhat disagree" or "disagree" in the above question:
	How did you enter the recipes that were not included in the options?	(Interviewer answered)
	I was able to add it myself as a new dish	Respondent was able to add it myself as a new dish
	I chose a similar dish	Respondent chose a similar dish
	I added/deleted foods of similar recipes	Respondent added/deleted foods of similar recipes
	I did not make an entry	Respondent did not make an entry
	Others	Others
**Identification of the amount you consumed** [c]	Were you able to make roughly accurate entries of the amount you consumed?	Were you able to accurately tell the telephone investigators of the amount you consumed?

AWARDJP, Automated Web-based assessment System Using Recipe-Data for Japanese.

[a] Self-administered web-based dietary 24-hour recall.

[b] Interviewer-administered telephone-based 24-hour recall.

[c] Select one option

[d] Select multiple options

eTable 3A. Questionnaire on AWARDJP acceptability: comparison by sex and age group in self-administered 24HR[a] (n=457)

	Total					<50 years					50–59 years					60–69 years					≥70 years				
	Men (n=292)		Women (n=165)		*P* value[c]	Men (n=86)		Women (n=62)		*P* value[c]	Men (n=112)		Women (n=71)		*P* value[c]	Men (n=67)		Women (n=25)		*P* value[c]	Men (n=27)		Women (n=7)		*P* value[c]
	n	%	n	%		n	%	n	%		n	%	n	%		n	%	n	%		n	%	n	%	
Ease of entry																									
Easy	27	10.8	13	8.6	0.824	11	14.7	7	11.7	0.731	10	10.4	6	9.7	0.362	6	10.3	0	0.0	0.412	0	0.0	0	0.0	1.000
Relatively easy	60	24.1	35	23.2		25	33.3	21	35.0		25	26.0	9	14.5		10	17.2	5	22.7		0	0.0	0	0.0	
Somewhat difficult	124	49.8	76	50.3		33	44.0	24	40.0		50	52.1	38	61.3		31	53.5	11	50.0		10	50.0	3	42.9	
Difficult	38	15.3	27	17.9		6	8.0	8	13.3		11	11.5	9	14.5		11	19.0	6	27.3		10	50.0	4	57.1	
Difficult part to enter[d]																									
Log in and select a date	11	6.8	6	5.8	0.755	3	7.7	2	6.3	1.000	1	1.6	4	8.5	0.165	3	7.1	0	0.0	0.550	4	20.0	0	0.0	0.545
Meal time and occasion	17	10.5	13	12.6	0.594	8	20.5	5	15.6	0.596	3	4.9	4	8.5	0.466	2	4.8	2	11.8	0.571	4	20.0	2	28.6	0.633
Dish name selection	116	71.6	74	71.8	0.966	28	71.8	18	56.3	0.172	44	72.1	38	80.9	0.293	34	81.0	14	82.4	1.000	10	50.0	4	57.1	1.000
Entering cooking details	91	56.2	71	68.9	0.038	17	43.6	23	71.9	0.017	40	65.6	31	66.0	0.967	24	57.1	12	70.6	0.338	10	50.0	5	71.4	0.408
Final confirmation screen	10	6.2	9	8.7	0.430	1	2.6	3	9.4	0.321	4	6.6	2	4.3	0.695	3	7.1	1	5.9	1.000	2	10.0	3	42.9	0.091
Others	12	7.4	6	5.8	0.618	3	7.7	1	3.1	0.622	5	8.2	2	4.3	0.697	2	4.8	1	5.9	1.000	2	10.0	2	28.6	0.269
Ease of food selection																									
Easy	30	22.1	15	17.7	0.746	14	31.8	6	20.7	0.547	11	18.0	8	19.5	0.442	5	19.2	1	7.7	0.526	0	0.0	0	0.0	1.000
Relatively easy	57	41.9	37	43.5		19	43.2	12	41.4		28	45.9	18	43.9		9	34.6	7	53.9		1	20.0	0	0.0	
Somewhat difficult	40	29.4	29	34.1		8	18.2	9	31.0		18	29.5	15	36.6		11	42.3	4	30.8		3	60.0	1	50.0	
Difficult	9	6.6	4	4.7		3	6.8	2	6.9		4	6.6	0	0.0		1	3.9	1	7.7		1	20.0	1	50.0	
Availability of dish options																									
Agree	47	35.1	27	31.8	0.192	19	43.2	13	40.6	0.931	21	35.0	11	28.2	0.045	7	26.9	3	25.0	0.690	0	0.0	0	0.0	1.000
Somewhat agree	59	44.0	34	40.0		18	40.9	12	37.5		29	48.3	14	35.9		10	38.5	7	58.3		2	50.0	1	50.0	
Somewhat disagree	22	16.4	23	27.1		6	13.6	6	18.8		8	13.3	14	35.9		7	26.9	2	16.7		1	25.0	1	50.0	
Disagree	6	4.5	1	1.2		1	2.3	1	3.1		2	3.3	0	0.0		2	7.7	0	0.0		1	25.0	0	0.0	
Alternative options when dishes were not available[e]																									
I was able to add it myself as a new dish	0	0.0	0	0.0	0.547	0	0.0	0	0.0	0.192	0	0.0	0	0.0	0.847	0	0.0	0	0.0	1.000	0	0.0	0	0.0	0.333
I chose a similar dish	10	35.7	10	41.7		0	0.0	2	28.6		3	30.0	6	42.9		5	55.6	2	100.0		2	100.0	0	0.0	
I added/deleted foods of similar recipes	2	7.1	4	16.7		0	0.0	1	14.3		1	10.0	2	14.3		1	11.1	0	0.0		0	0.0	1	100.0	
I did not make an entry	1	3.6	0	0.0		0	0.0	0	0.0		0	0.0	0	0.0		1	11.1	0	0.0		0	0.0	0	0.0	
Others	0	0.0	0	0.0		0	0.0	0	0.0		0	0.0	0	0.0		0	0.0	0	0.0		0	0.0	0	0.0	
Identification of the amount you consumed																									
Agree	57	48.3	45	55.6	0.477	20	47.6	20	66.7	0.317	26	51.0	20	54.1	0.481	8	38.1	5	41.7	1.000	3	75.0	0	0.0	0.200
Somewhat agree	44	37.3	23	28.4		17	40.5	7	23.3		19	37.3	10	27.0		8	38.1	5	41.7		0	0.0	1	50.0	
Somewhat disagree	16	13.6	13	16.1		4	9.5	3	10.0		6	11.8	7	18.9		5	23.8	2	16.7		1	25.0	1	50.0	
Disagree	1	0.9	0	0.0		1	2.4	0	0.0		0	0.0	0	0.0		0	0.0	0	0.0		0	0.0	0	0.0	

24HR, 24-hour dietary recall; AWARDJP, Automated Web-based assessment System Using Recipe-Data for Japanese; N/A, not applicable.
The "total" percentage is expressed as a percentage of the total number of participants. The percentage of men/women responses is expressed as the percentage of the total number of those who used each method.
Missing values are excluded for each item.
[a] Self-administered web-based dietary 24-hour recall.
[b] Interviewer-administered telephone-based 24-hour recall.
[c] $\chi 2$ test: Fisher's exact test (direct method) is used when the number of cells with expected values less than 5 exceeded 20% of the total.
[d] The denominator is the number of subjects who selected "Somewhat difficult" or "Difficult" in the above question.
[e] The denominator is the number of subjects who selected "Somewhat disagree" or "Disagree" in the above question.

eTable 3B. Questionnaire on AWARDJP acceptability: comparison by sex and age group in interviewer-administered 24HR[b] (n=518)

	Total					<50 years					50–59 years					60–69 years					≥70 years				
	Men (n=179)		Women (n=339)		*P* value[c]	Men (n=12)		Women (n=22)		*P* value[c]	Men (n=19)		Women (n=60)		*P* value[c]	Men (n=56)		Women (n=121)		*P* value[c]	Men (n=92)		Women (n=136)		*P* value[c]
	n	%	n	%		n	%	n	%		n	%	n	%		n	%	n	%		n	%	n	%	
Ease of entry																									
Easy	103	62.4	210	67.1	0.277	8	72.7	14	70.0	1.000	13	68.4	42	76.4	0.73	39	76.5	75	67.0	0.335	43	51.2	79	62.7	0.096
Relatively easy	41	24.9	77	24.6		2	18.2	5	25.0		4	21.1	9	16.4		7	13.7	27	24.1		28	33.3	36	28.6	
Somewhat difficult	16	9.7	23	7.4		1	9.1	1	5.0		2	10.5	4	7.3		5	9.8	8	7.1		8	9.5	10	7.9	
Difficult	5	3.0	3	1.0		0	0.0	0	0.0		0	0.0	0	0.0		0	0.0	2	1.8		5	6.0	1	0.8	
Difficult part to enter[d]																									
Log in and select a date	N/A		N/A			N/A		N/A			N/A		N/A			N/A		N/A			N/A		N/A		
Meal time and occasion	0	0.0	1	3.8	1.000	0	0.0	0	0.0	N/A	0	0.0	0	0.0	N/A	0	0.0	1	10.0	1.000	0	0.0	0	0.0	N/A
Dish name selection	9	42.9	4	15.4	0.036	1	100.0	0	0.0	1.000	0	0.0	1	25.0	1.000	3	60.0	1	10.0	0.077	5	38.5	2	18.2	0.386
Telling cooking details	7	33.3	12	46.2	0.373	1	100.0	1	100.0	N/A	1	50.0	2	50.0	1.000	2	40.0	3	30.0	1.000	3	23.1	6	54.5	0.206
Final confirmation screen	0	0.0	0	0.0	N/A	0	0.0	0	0.0	N/A	0	0.0	0	0.0	N/A	0	0.0	0	0.0	N/A	0	0.0	0	0.0	N/A
Others	5	23.8	7	26.9	0.808	0	0.0	0	0.0	N/A	0	0.0	1	25.0	1.000	2	40.0	4	40.0	1.000	3	23.1	2	18.2	1.000
Ease of food selection																									
Agree	120	80.5	234	78.8	0.577	7	70.0	16	84.2	0.633	16	94.1	43	82.7	0.432	39	84.8	79	75.2	0.192	58	76.3	96	79.3	0.607
Somewhat agree	27	18.1	60	20.2		3	30.0	3	15.8		1	5.9	9	17.3		7	15.2	26	24.8		16	21.1	22	18.2	
Somewhat disagree	1	0.7	3	1.0		0	0.0	0	0.0		0	0.0	0	0.0		0	0.0	0	0.0		1	1.3	3	2.5	
Disagree	1	0.7	0	0.0		0	0.0	0	0.0		0	0.0	0	0.0		0	0.0	0	0.0		1	1.3	0	0.0	
Availability of dish options																									
Agree	109	68.6	182	61.7	0.388	6	60.0	13	68.4	1.000	11	64.7	38	73.1	0.789	32	65.3	69	65.7	0.949	60	72.3	62	52.1	0.020
Somewhat agree	42	26.4	92	31.2		3	30.0	5	26.3		5	29.4	11	21.2		16	32.7	32	30.5		18	21.7	44	37.0	
Somewhat disagree	7	4.4	20	6.8		1	10.0	1	5.3		1	5.9	3	5.8		1	2.0	4	3.8		4	4.8	12	10.1	
Disagree	1	0.6	1	0.3		0	0.0	0	0.0		0	0.0	0	0.0		0	0.0	0	0.0		1	1.2	1	0.8	
Alternative options when dishes were not available[e]																									
I was able to add it myself as a new dish	0	0.0	0	0.0	0.753	0	0.0	0	0.0	N/A	0	0.0	0	0.0	0.250	0	0.0	0	0.0	0.200	0	0.0	0	0.0	0.869
I chose a similar dish	2	25.0	4	19.0		0	0.0	0	0.0		0	0.0	0	0.0		0	0.0	0	0.0		2	40.0	4	30.8	
I added/deleted foods of similar recipes	2	25.0	9	42.9		0	0.0	0	0.0		1	100.0	0	0.0		0	0.0	4	100.0		1	20.0	5	38.5	
I did not make an entry	0	0.0	0	0.0		0	0.0	0	0.0		0	0.0	0	0.0		0	0.0	0	0.0		0	0.0	0	0.0	
Others	0	0.0	1	4.8		0	0.0	0	0.0		0	0.0	0	0.0		0	0.0	0	0.0		0	0.0	1	7.7	
Identification of the amount you consumed																									
Agree	128	78.5	249	80.1	0.735	8	72.7	18	90.0	0.317	14	73.7	47	85.5	0.193	40	80.0	85	77.3	0.796	66	79.5	99	78.6	0.923
Somewhat agree	31	19.0	52	16.7		3	27.3	2	10.0		5	26.3	6	10.9		9	18.0	23	20.9		14	16.9	21	16.7	
Somewhat disagree	3	1.8	9	2.9		0	0.0	0	0.0		0	0.0	2	3.6		0	0.0	1	0.9		3	3.6	6	4.8	
Disagree	1	0.6	1	0.3		0	0.0	0	0.0		0	0.0	0	0.0		1	2.0	1	0.9		0	0.0	0	0.0	

24HR, 24-hour dietary recall; AWARDJP, Automated Web-based assessment System Using Recipe-Data for Japanese; N/A, not applicable.
The "total" percentage is expressed as a percentage of the total number of participants. The percentage of men/women responses is expressed as the percentage of the total number of those who used each method.
Missing values are excluded for each item.

[a] Self-administered web-based dietary 24-hour recall.

[b] Interviewer-administered telephone-based 24-hour recall.

[c] χ2 test: Fisher's exact test (direct method) is used when the number of cells with expected values less than 5 exceeded 20% of the total.

[d] The denominator is the number of subjects who selected "Somewhat difficult" or "Difficult" in the above question.

[e] The denominator is the number of subjects who selected "Somewhat disagree" or "Disagree" in the above question.
